# Successful smoking cessation with electronic cigarettes in smokers with a documented history of recurring relapses: a case series

**DOI:** 10.1186/1752-1947-5-585

**Published:** 2011-12-20

**Authors:** Pasquale Caponnetto, Riccardo Polosa, Cristina Russo, Carmelo Leotta, Davide Campagna

**Affiliations:** 1 Centro per la Prevenzione e Cura del Tabagismo (CPCT), Dipartimento di Biomedicina Clinica e Molecolare, Azienda Ospedaliero-Universitaria "V. Emanuele-Policlinico", Università di Catania, Catania, Italy; 2 Institute of Internal Medicine, S. Marta Hospital, Azienda Ospedaliero-Universitaria "V. Emanuele-Policlinico", Università di Catania, Catania, Italy; 3 Unità Operativa Geriatria, Dipartimento di Chirurgia Sessione Geratria, Ospedale Cannizzaro, Università di Catania, Catania, Italy

## Abstract

**Introduction:**

Smoking cessation programs are useful in helping smokers to quit, but smoking is a very difficult addiction to break and the need for novel and effective approaches to smoking cessation interventions is unquestionable. The E-cigarette is a battery-powered electronic nicotine delivery device that may help smokers to remain abstinent during their quit attempt. We report for the first time objective measures of smoking cessation in smokers who experimented with the E-cigarette.

**Case presentation:**

Three Caucasian smokers (two men aged 47 and 65 years and one woman aged 38 years) with a documented history of recurring relapses were able to quit and to remain abstinent for at least six months after taking up an E-cigarette.

**Conclusions:**

This is the first time that objective measures of smoking cessation are reported for smokers who quit successfully after using an E-cigarette. This was accomplished in smokers who repeatedly failed in previous attempts with professional smoking cessation assistance using the usual nicotine dependence treatments and smoking cessation counselling.

## Introduction

Cigarette smoke harms nearly every system of the human body, thus causing a broad range of diseases, many of which are fatal [1,2]. The risk of serious disease diminishes rapidly after quitting and life-long abstinence is known to reduce the risk of lung cancer, heart disease, strokes, chronic lung disease and other cancers [3,4]. Although evidence-based recommendations indicate that smoking cessation programs are useful in helping smokers to quit [5], smoking is a very difficult addiction to break. It has been shown that approximately 80% of smokers who attempt to quit on their own relapse within the first month of abstinence and only about 3% to 5% remain abstinent at six months [6]. Although there is little doubt that currently-marketed smoking cessation products increase the chance of committed smokers stopping smoking, they reportedly lack high levels of efficacy - particularly in the real life setting [7]. Although this is known to reflect the chronic relapsing nature of tobacco dependence, the need for novel and effective approaches to smoking cessation interventions is unquestionable.

**Figure 1:**
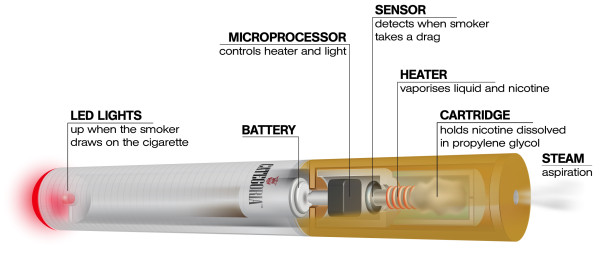
**The E-cigarette is a battery-powered electronic nicotine delivery device (ENDD) designed for the purpose of providing inhaled doses of nicotine by way of a vaporized solution to the respiratory system**. This device provides a flavor and physical sensation similar to that of inhaled tobacco smoke, while no smoke or combustion is actually involved in its operation. It is composed of the following key components: **(1) **the inhaler - also known as 'cartridge' (a disposable plastic mouthpiece - resembling a tobacco cigarette's filter - which contains an absorbent material that is saturated with a liquid solution containing nicotine); **(2) **the atomizing device (the heating element that vaporizes the liquid in the mouthpiece and generates the mist with each puff); **(3) **the battery component (the body of the device - resembling a tobacco cigarette - which houses a lithium-ion re-chargeable battery to power the atomizer). The body of the device also houses an electronic airflow sensor to automatically activate the heating element upon inhalation and to light up a LED (Light Emitting Diode) indicator to signal activation of the device with each puff.

The E-cigarette is a battery-powered electronic nicotine delivery device (ENDD), often resembling a cigarette. It is designed to deliver nicotine to the respiratory system, where neither tobacco nor combustion are necessary for its operation [8] (Figure 1). Consequently, it is likely that this product may be considered as a lower risk substitute for factory-made cigarettes. In addition, people report buying them to help quit smoking, to reduce cigarette consumption, and to relieve tobacco withdrawal symptoms due to workplace smoking restrictions [9]. Besides delivering nicotine to the lung, E-cigarettes may also provide a coping mechanism for conditioned smoking cues by replacing some of the rituals associated with smoking gestures (for example the hand-to-mouth action of smoking). For this reason, E-cigarettes may help smokers to remain abstinent during their quit attempt. To date there has been no formal demonstration supporting the efficacy of these devices in smoking cessation studies.

To the best of our knowledge, we report for the first time objective measures of smoking cessation in three heavy smokers who experimented with the E-cigarette.

## Case presentations

In this case series we describe three heavy smokers with an established history of relapses who have been repetitively managed for nicotine dependence at our university clinic for smoking cessation (Centro per la Prevenzione e Cura del Tabagismo - CPCT; Università di Catania; Italy). Our patients (two men aged 47 and 65 years and one woman aged 38 years), were of Caucasian ethnicity. At CPCT, smoking cessation programs are based on an adaptation of the Clinical Practice Guideline on Smoking Cessation of the U.S. Department of Health and Human Services [5] and have been described previously in detail [10]. The staff at CPCT includes a dedicated team of clinical psychologists, physicians, and nurses with at least three years of experience.

### Patient 1

A 47-year-old Caucasian male lawyer with a diagnosis of severe nicotine dependence attended our smoking cessation clinic four years ago. He smoked 32 cigarettes per day (45 pack/years) with a significant level of nicotine dependence (Fagerstrom Test of Nicotine Dependence - FTND = 8). His concentration of exhaled breath carbon monoxide (eCO) reading at baseline was 31 ppm. No history of alcohol abuse, major depression or other psychiatric conditions was reported. He was subjected to intensive treatment for nicotine dependence four years ago and subsequently after seven months. He participated in other intensive treatments for nicotine dependence three years ago and two years ago. On each occasion, he was prescribed a combination of nicotine patches and bupropion and was offered smoking cessation counselling throughout the program. His last relapse occurred one month after treatment'. During a routine telephone follow-up two years ago, he reported having quit smoking on his own after taking up an E-cigarette. He was then invited to visit our clinic to allow us to collect more informations and conduct further investigations. He told us that he started experimenting with an E-cigarette (loaded with a high nicotine concentration: 7.2 mg nicotine per cartridge) two years ago. A few weeks later, he was able to discontinue tobacco smoking completely. He kept using his E-cigarette for another few months before stopping use of the E-cigarette as well. Abstinence from tobacco smoking was then objectively assessed by measuring the concentration of exhaled breath carbon monoxide concentration (eCO); the measured eCO value was within the normal range (eCO = 4 ppm). He has been abstinent from tobacco smoking for approximately six months with no reported lapse or relapse during this period of time. The E-cigarette was well tolerated with no reported adverse effects.

### Patient 2

A 38-year-old Caucasian female social worker with a diagnosis of severe nicotine dependence attended our smoking cessation clinic four years ago. She smoked 28 cigarettes per day (28 pack/years) with a significant level of nicotine dependence (FTND = 8). Her eCO reading at baseline was 29 ppm. Some mild depression assessed by the Self-rating Depression Scale (SDS) was also documented in her case notes. She was treated for nicotine dependence at our clinic four years ago and again two years ago. On each occasion, she was prescribed nicotine patches and bupropion. She was offered smoking cessation counselling throughout the program. Her last relapse occurred two years ago.

During a routine telephone follow-up one year ago, she reported having quit smoking on her own after taking up an E-cigarette. She was then invited for a follow-up visit at our clinic, during which abstinence was reviewed objectively by measuring the concentration of eCO. She told us that she had started experimenting with an E-cigarette (loaded with high nicotine concentration: 7.2 mg nicotine per cartridge) two years ago. Three months later, she was able to discontinue tobacco smoking completely. She kept using the E-cigarette with a high nicotine concentration for another month before switching to mentholated cartridges, which she now uses frequently during social events.

Abstinence from tobacco smoking was confirmed objectively by very low levels of eCO (eCO = 2 ppm). She has been abstinent from tobacco smoking for approximately seven months with no reported lapse or relapse during this period of time. Overall, the E-cigarette was well tolerated with occasional dry cough being reported.

### Patient 3

A 65-year-old Caucasian male pharmacist with a known diagnosis of chronic obstructive pulmonary disease (COPD) was seen for a routine follow up in our chest clinic two years ago. He had been a heavy smoker for nearly 50 years (89 pack/years) and had a past history of alcohol abuse. He was treated for nicotine dependence twice by the local services for addiction on two occasions seven years ago and four years ago. On both occasions he was prescribed nicotine patches and attended group counselling sessions. Four years ago, he came to our smoking cessation clinic. He smoked 30 to 40 cigarettes per day with a significant level of nicotine dependence (FTND = 10). His eCO baseline reading was 34.9 ppm. He was started on varenicline (a partial agonist of the α4β2 nicotinic acetylcholine receptor approved specifically for smoking cessation therapy) in association with smoking cessation counselling, but he was lost to follow-up by one month after his quit date.

When he came for his routine follow-up appointment at the chest clinic two years ago, he announced that he had quit tobacco smoking on his own after taking up an E-cigarette loaded with nicotine cartridges. Two months after taking up an E-cigarette loaded with nicotine cartridges, he was able to discontinue tobacco smoking completely. He continued using his E-cigarette on a regular basis. Predictably, this patient noted a significant improvement in quality of life, manifested by increased energy levels and exercise tolerance. Moreover, he has reported no significant exacerbations of his symptoms during the past two years.

Abstinence from tobacco smoking was confirmed objectively by measuring the concentration of exhaled breath carbon monoxide concentration (eCO); the measured eCO value being within the normal range (eCO = 5 ppm). The E-cigarette was well tolerated with no reported adverse events.

## Discussion

This is the first time that objective measures of smoking cessation are reported in smokers with a documented history of recurring relapses, who quit smoking after taking up an E-cigarette with the intention of quitting tobacco smoking. This was accomplished by heavy smokers who repeatedly failed in previous attempts with professional smoking cessation assistance based on the usual nicotine dependence treatments and smoking cessation counselling. Some studies have found that multiple failed attempts have a negative effect on a smoker's confidence in being able to quit smoking cigarettes [11].

The COPD patient was a particularly difficult case with an FTND of 10 (maximum score) and a documented history of recurring relapses. The available evidence in the medical literature indicates that, in contrast with smokers in the general population, COPD patients who smoke typically respond poorly to smoking cessation efforts; they have a greater degree of physical nicotine dependence [12] and appear to be less motivated to quit smoking [13].

We cannot discount that the success observed in our patients may be simply due to the repeated number of quit attempts and not necessarily to E-cigarette use. However, we later contacted these patients and asked if they took up the E-cigarette with the intention to quit and if they believed that they would not have quit if it weren't for the E-cigarettes. Answers to both questions were positive for all three patients. Thus, these patients felt that they would not have quit tobacco smoking without the help of E-cigarettes.

The remarkable success stories of these three smokers require consideration. The widely acknowledged beneficial role of pharmacotherapy in smoking cessation is likely to be due to its ability to address the physical component of tobacco dependence. However, taking pills or patches for nicotine addiction is unlikely to resolve the psychological components associated with tobacco dependence. As a matter of fact smoking is much more than the addicting effect of nicotine; the smoking habit also includes the rituals that each smoker associates with his or her habit [14]. Smoking cessation products cannot replace the rituals associated with the act of smoking.

Counselling for smoking cessation is intended to help smokers in coping with this important aspect of their life by implementing personalized replacement rituals, but even counselling for smoking cessation lacks high levels of efficacy. Therefore, it is likely that the smokers described in our case series coped successfully with the psychological components associated with their tobacco dependence by using a device resembling a cigarette, which - although being mainly designed for the purpose of nicotine delivery to the respiratory system - has the additional advantage of being a valid substitute for the tactile sensations of the cigarette and other sensations associated with smoking gestures.

An important aspect that needs to be highlighted in relation to the findings of the present case series is the putative risk of E-cigarettes. In June 2009, the US Food and Drug Administration (FDA) announced in a press conference that 'a laboratory analysis of electronic cigarette samples has found that they contain carcinogens and toxic chemicals such as diethylene glycol (DEG), an ingredient used in antifreeze' [15]. The actual lab report revealed that the 'carcinogens' referred to in the FDA's press conference were tobacco specific nitrosamines (TSNAs), but failed to specify the quantity detected. The FDA's report did state that the quantity of DEG detected in the liquid in one of the 18 samples was 1% (0.01 ml), but did not point out that this is a non-toxic quantity. The FDA did not report finding DEG, or any other harmful chemical, in the vapor [16]. A number of reports available over the Internet have subsequently characterized, quite extensively, the components contained in E-cigarette liquid and vapor using gas chromatography mass spectrometry (GC-MS). They demonstrate that the primary components of E-cigarette cartridges are propylene glycol (PG), glycerin, and nicotine [17]. Laugesen tested E-cigarette mist for more than 50 priority-listed cigarette smoke toxicants and found none [18]. This report only revealed traces (8.2 ng/g) of TSNAs in the 'high' nicotine cartridge of an E-cigarette.

It must be noted that this amount is equal to the quantity reported to be present in a nicotine medicinal patch.

Recently, Cahn and Siegel have reviewed the results of 16 laboratory analyses of E-cigarette liquid, including the FDA's 'Final Report'. TSNAs were reported in two studies, but at trace levels, which are similar to those found in a nicotine patch, and, most importantly, about 500-fold to 1400-fold lower than TSNA levels measured in regular cigarettes. The presence of DEG was reported in the FDA's report in one of the 18 cartridges, yet none of the other 15 studies found any DEG. The authors stated, 'Other than TSNAs and DEG, few, if any, chemicals at levels detected in E-cigs raise serious health concerns. Although the current data are insufficient to conclude that E-cigarettes are safe in absolute terms and that further studies are needed to comprehensively assess their safety, these products appear to be much safer than tobacco cigarettes and comparable in toxicity to conventional nicotine replacement products' [19].

In a recent prospective proof-of-concept study, we monitored possible modifications in the smoking habits of 40 smokers not willing to quit who were experimenting with a 7.4 mg nicotine/cartridge E-cigarette [20]. Combined sustained smoking reduction and smoking abstinence was shown in 55% of the participants, with an overall 88% fall in the number of cigarettes smoked per day. Mouth and throat irritation, and dry cough were common, but diminished substantially by the end of the study. Retailers all over the world have already sold hundreds of thousands of E-cigarettes, yet there is no evidence that these products have endangered anyone.

Lastly, there may be some concern that non-smokers might take up use of an E-cigarette, become addicted to nicotine, and eventually start to smoke tobacco cigarettes. The fear of this 'gateway effect' has been mentioned in connection with the European Union ban on the sale of snus, a type of smokeless tobacco that is neither chewed nor smoked. The available evidence would indicate that snus provides a gateway out of smoking rather than into it. Snus is a type of finely ground moist snuff that delivers significant levels of nicotine. Snus does not produce any of the toxic combustion products and it is manufactured in a way that produces low levels of tobacco-specific nitrosamines, the main carcinogens responsible for oral cancers in users of other smokeless tobacco products [21].

Sweden now has one of the lowest smoking prevalence rates in the world [22]. Ranstrom and Foulds found the odds of initiating daily smoking were significantly lower for men who had started using snus than for those who had not (odds ratio (OR) = 0.28, 95% confidence interval (CI) 0.22 to 0.36) [23]. Another study found that the quit ratio for smoking was significantly higher for daily snus users in six of seven data sets collected during 2003 to 2008 in Norway [24]. In the United States of America (USA) 73% of the most recent quit attempts using smokeless tobacco resulted in smokers achieving smoking abstinence [25].

In a survey that included 3037 ever-users of E-cigarettes, only one of the 2850 respondents who used nicotine-containing E-cigarettes was a never-smoker [26]. The authors of the study did not report the reason. Given the fact that 70% of the ever-users succeeded in quitting smoking, the E-cigarette would also appear to be a gateway away from smoking. In previous quit attempts, 70.5% had tried nicotine therapy, 29.1% used bupropion, and 19.4% used varenicline. Users of both snus and E-cigarettes might be less likely to later switch to smoking if governments and health organizations made it clear that smoking carries enormously greater health risks than nicotine that comes from non-smoked sources.

Obviously, these products need to be adequately regulated, but thus far, there have been heterogeneous regulatory responses. Some countries have completely banned the sale and marketing of E-cigarettes whereas others allow marketing within their regulatory frameworks. Internet marketing of E-cigarettes and the inadequacy and misapplication of import product codes, however, impede systematic regulation [27]. More research on E-cigarettes must be conducted in order to ensure that the decisions of regulators, healthcare providers and consumers are evidence-based.

## Conclusions

The most important message from this case series is that these smokers, with a documented history of recurring relapses, were able to quit smoking and to remain abstinent for at least six months after taking up an electronic cigarette. Although the present findings cannot be generalized, high quit rates would be desirable in a population that generally responds poorly to smoking cessation efforts. Larger controlled studies are needed to confirm this interesting finding, particularly for those smokers for whom the handling and manipulation of their cigarettes play an important part of the ritual of smoking.

## Consent

Written informed consent was obtained from the patients for publication of this case report and any accompanying images. A copy of the written consent is available for review by the Editor-in-Chief of this journal.

## Competing interests

The authors declare that they have no competing interests.

## Authors' contributions

RP, PC, CR, CL and DC were responsible for the study conception, data retrieval and draft of the manuscript. All authors read and approved the final manuscript.
